# Modelling Cumulative Seismic Damage at the Urban Scale

**DOI:** 10.3390/e28070807

**Published:** 2026-07-15

**Authors:** Rosa Maria Sava, Annalisa Greco, Alessandro Pluchino, Andrea Rapisarda

**Affiliations:** 1 Department of Physics and Astronomy “Ettore Majorana”, University of Catania, Via Santa Sofia 64, 95123 Catania, Italy; alessandro.pluchino@dfa.unict.it (A.P.); andrea.rapisarda@dfa.unict.it (A.R.); 2 INFN Sezione di Catania, Via Santa Sofia 64, 95123 Catania, Italy; 3 Department of Civil-Industrial Engineering and Architecture, University of Catania, Via Santa Sofia 64, 95123 Catania, Italy; annalisa.greco@unict.it; 4 Complexity Science Hub, Metternichgasse 8, 1030 Vienna, Austria

**Keywords:** seismic sequences, buildings vulnerability, damage scenarios at urban scale

## Abstract

The analysis of earthquake-induced damage scenarios at the urban scale is a fundamental tool for seismic risk assessment and mitigation and the management of urbanized areas exposed to seismic hazards. This paper presents a methodology for simulating earthquake damage scenarios over large urban territories that explicitly accounts for the cumulative effects of seismic sequences. The proposed approach models the progressive accumulation of structural damage and the resulting evolution of building vulnerability under repeated seismic loading. From a complex systems perspective, the methodology describes urban areas as collections of buildings whose vulnerability evolves through memory-dependent processes. Under this framework, the final damage scenario emerges from the cumulative effects of the entire seismic history rather than from the contribution of individual earthquakes considered in isolation. The study extends previous work by the authors, in which instrumentally derived macroseismic intensity maps were integrated with observed building damage data from the 2009 L’Aquila seismic sequence. The results demonstrated that the methodology could successfully reproduce the spatial distribution of observed damage when considering not only the mainshock but also all seismic events exceeding a selected magnitude threshold. In this contribution, new developments of the calibration procedure are presented, together with applications to the 2013 Garfagnana-Lunigiana and the 2016–2017 Central Italy seismic sequences. Through a comparative analysis of these case studies, the influence of different seismic sequence characteristics and building stock features on damage evolution is investigated. The results provide further insight into the capabilities and limitations of the proposed methodology, highlighting its potential as a tool for interpreting post-earthquake damage patterns and supporting seismic risk assessment and mitigation strategies.

## 1. Introduction

Urban-scale seismic damage scenario analysis is a fundamental tool for seismic risk assessment, mitigation planning, and emergency management. By integrating information on seismic hazard, exposure, and vulnerability, it supports the development of effective prevention measures, preparedness strategies, and post-earthquake response actions. A major challenge in this context is the proper treatment of seismic sequences, during which buildings are subjected to repeated ground shaking. Such repeated loading progressively degrades structural capacity, causing damage states that result from cumulative effects rather than from a single seismic event. More generally, cumulative seismic damage can be interpreted as a complex system phenomenon. Urban building stocks constitute heterogeneous systems composed of individual units characterized by different structural properties and vulnerability levels. Repeated seismic forcing progressively modifies the state of each unit, introducing memory effects and path-dependent dynamics. As a consequence, the final damage distribution observed after a seismic sequence can be regarded as an emergent property arising from the nonlinear interaction between seismic excitation and vulnerability evolution.

This issue has been extensively investigated from several complementary perspectives. Research efforts have focused on the development of models capable of forecasting the spatial and temporal clustering of earthquakes within seismic sequences [1,2]; on methodologies aimed at evaluating the structural characteristics that govern building vulnerability, including simplified building models [3,4,5], Vulnerability Index Methods [6], as well as external factors that may contribute to vulnerability evolution over time [7,8]; and on approaches for estimating expected damage as a function of both building vulnerability and seismic intensity. These include Damage Probability Matrices [9], vulnerability and fragility curves [10,11,12,13,14], and more recently, Machine Learning techniques [15]. Particular attention has also been devoted to modelling cumulative damage processes. Within the framework of performance-based earthquake engineering, several numerical studies have investigated the response of different construction typologies subjected to repeated seismic loading [16,17,18,19,20]. In parallel, a variety of software tools have been developed for seismic damage scenario simulation ([21] and references therein). Only recently, however, has the first software specifically designed for state-dependent seismic damage assessment been introduced [22], highlighting the growing importance of cumulative damage modelling in seismic risk analysis.

The simulation of future damage scenarios is intrinsically connected to the study of damage patterns observed during past earthquakes. In historical seismology, considerable effort has been devoted to the interpretation of historical sources in order to distinguish the effects attributable to individual earthquakes within complex seismic sequences [23]. More recently, dedicated methodologies have been proposed to investigate the influence of cumulative seismic intensity on the estimation of source parameters [24,25]. These issues are particularly relevant because the seismic parameters reported in historical catalogues, such as CFTI5Med [26,27] and CPTI [28,29], constitute a key basis for seismic hazard assessment.

In Italy, damage data from recent and contemporary earthquakes are collected through field surveys coordinated by the Department of Civil Protection, with the support of expert teams from research institutions, e.g., the Quick Earthquake Survey Team (QUEST). A substantial portion of these observations has been organized within the Database of Observed Damage (Da.D.O.), a WebGIS platform developed by Eucentre [30,31] to provide researchers with homogeneous and standardized datasets. Over the years, different operational survey procedures have been adopted, culminating in the introduction of the AeDES form [32,33]. The Da.D.O. database contains extensive information on the damage recorded across large building stocks affected by the most significant Italian seismic sequences, from the 1976 Friuli earthquakes to the 2019 Mugello event. However, the available information generally describes the final damage state reached at the end of a seismic sequence, without documenting the intermediate stages of damage progression. The first Italian case in which damage surveys were repeated after successive earthquakes occurred during the 2016–2017 Central Italy seismic sequence [34]. These surveys highlighted how the large variability in building characteristics, pre-existing conditions, and local environmental factors makes both damage assessment and macroseismic intensity evaluation particularly challenging.

Developing models that explicitly account for all these aspects, together with the detailed structural response of every building affected by a seismic sequence, would require prohibitive computational resources. To address this issue, Greco et al. [35] and Fischer et al. [36] proposed a simplified methodology for simulating the effects of seismic sequences over large urban areas by modelling both damage accumulation and vulnerability evolution. Their approach employed an agent-based model based on a self-organized criticality engine to reproduce a seismic sequence similar to that of L’Aquila 2009 and applied it to the urban areas of Avola and Catania, in Sicily. Building upon this framework, Sava et al. [37] introduced an alternative vulnerability evolution function and calibrated the model parameters using observed seismic sequence data and corresponding damage scenarios.

In this paper, Section 2 briefly summarizes the methodology proposed in [37] and introduces the developments presented in the current study. Section 3 reports the results of the calibration performed using the 2009 L’Aquila seismic sequence. Section 4 and Section 5 extend the analysis to the 2013 Garfagnana-Lunigiana and the 2016–2017 Central Italy seismic sequences, respectively, which are adopted as additional case studies. Finally, Section 6 discusses the similarities and differences emerging from the three applications, highlighting both the strengths and the current limitations of the proposed methodology, while Section 7 closes the paper.

## 2. Methodology: Simulations of Seismic Damage Accumulation Scenario

In this study, we further investigate an enhanced version of the methodology presented in [37] for the simulation of cumulative seismic damage scenarios in urban areas. Building upon the framework originally introduced in [35,36], the previous work numerically reproduced the cumulative effects of seismic sequences on urban building stocks according to the following assumptions: (a) each earthquake in a seismic sequence contributes to the accumulation of structural damage, progressively increasing building vulnerability; (b) as vulnerability increases, subsequent earthquakes, regardless of whether they are weaker or stronger than preceding events, may induce higher levels of damage than would otherwise be expected; and (c) the final damage state observed at the end of the sequence results from the cumulative contribution of all individual damage increments produced by the earthquakes composing the sequence.

In [37], the methodology was applied to the 2009 L’Aquila seismic sequence. The seismic input was represented through instrumentally derived macroseismic intensity maps (ShakeMaps) produced by the Italian National Institute of Geophysics and Volcanology (INGV) [38,39]. Among all recorded earthquakes, only the eighteen events with magnitude Mw ≥ 4.0 occurring between the Mw 6.1 mainshock of 6 April 2009 and the Mw 5.0 event of 9 April 2009 were considered. The building stock was reconstructed using data from the post-earthquake surveys available in the Da.D.O. [30,31], which were employed both to define the initial conditions of each building (location within the ShakeMaps and initial vulnerability class) and to compare the simulated damage distributions with the observed ones. The analysis focused exclusively on masonry buildings, as they constitute the most representative category within the available dataset.

Damage was classified according to the discrete scale ranging from D0 (no damage) to D5 (collapse), with intermediate levels D1 (light damage), D2 (moderate damage), D3 (severe damage), and D4 (very severe damage). The initial vulnerability of masonry buildings was instead categorized into classes ranging from A (most vulnerable) to C1 (least vulnerable), according to the structural characteristics of both vertical and horizontal elements and the presence of chains, as summarized in Table 1. To estimate damage after each earthquake and to assign the corresponding initial numerical vulnerability values (Table 1), we adopted the fragility model proposed in [13], hereafter referred to as the “L21” model:

(1) $$\mu_{D}=\left\{\begin{array}{c} 2.5\;\left[1+\tanh\left(\frac{I+3.45V-11.7}{0.9+2.8V}\right)\right]\;for\;V\geq 0.32\\ 2.5\;\left[1+\tanh\left(\frac{I+6.25V-12.6}{1.8}\right)\right]\;for\;V\;<0.32. \end{array}\right.$$

Schematically, for each earthquake *i* of a sequence of *N* events: the damage level ${DL}_{i}$ is calculated as the most probable damage from the L21 model, as a function of the intensity $I_{i}$ of the event and of the vulnerability $V_{i-1}$ of the building, that is the updated vulnerability after the previous event: ${DL}_{i}=f\left(V_{i-1},\;I_{i}\right)$, i=1,…N. The total damage level ${TDL}_{i}$ is calculated as the sum of all the damage levels up the *i*-th event included: ${TDL}_{i}=\sum_{j=1}^{i}{DL}_{j}$. Finally, the vulnerability is updated as a function of the initial vulnerability $V_{0}$ and of the total damage level reached after the *i*-th event: $V_{i}={g(V}_{0},\;{TDL}_{i})$. For the first event, the damage level is calculated as a function of the initial vulnerability ${DL}_{1}=f\left(V_{0},\;I_{1}\right)$ and ${TDL}_{1}={DL}_{1}$, assuming all buildings start from an undamaged condition. As a vulnerability update rule, ref. [37] introduced an exponential function (Equation (2)), hereafter referred to as “Exp”, together with a new vulnerability class A-, defined within the interval [1.15, 1.25]. This additional class represents buildings whose vulnerability has increased beyond their original classification as a consequence of damage accumulation.

(2) ViEXP=V0+maxA−−V0ekTDLi−3−e−3ke2k−e−3k, k=1V0

After the last earthquake of the sequence, the cumulative damage value assigned to each building is converted into a categorical damage class ranging from D0 to D5. To ensure consistency in the comparison between simulations and observations, we also considered the global observed damage grade, *D_Global_*, introduced in [13]. This quantity represents a weighted average of the damage observed in five structural components (vertical structures, horizontal structures, stairs, roofs, and infills), based on the survey information available in the Da.D.O. database.

The discrepancy between simulated and observed damage distributions was quantified through the Root Mean Squared Error (RMSE), defined as:

(3) $$RMSE=\;\sqrt{\frac{1}{6}\sum_{i=0}^{5}{\left(F_{i}^{obs}-F_{i}^{sim}\right)}^{2}}$$

where $F_{i}^{obs}$ and $F_{i}^{sim}$ represent the observed and simulated percentage of buildings, respectively, associated with each final damage class from D0 to D5.

To account for sources of variability not explicitly included in the model, randomness was introduced at two levels. First, the initial vulnerability value $V_{0}$ of each building was randomly assigned within the plausible range associated with its vulnerability class (Table 1). Second, for each earthquake, only a fixed percentage of buildings was randomly selected to be affected by the seismic input. To implement this procedure, each damage scenario was simulated over multiple realizations (*N.seeds*). For every earthquake, a fraction *p* of the buildings corresponding to each ShakeMap intensity value was randomly sampled. We recall that each earthquake of the sequence is represented in our model by the associated ShakeMap generated for the given event by the INGV in the multipolygon shapefile format. Each polygon is characterized by a unique value of the intensity, which represents the instrumentally derived intensity felt at the area included in the polygon (considering amplification or deamplification phenomena). Therefore, the *p* parameter represents the percentage of buildings randomly selected among those located within each ShakeMap polygon; that is, among those with the same value of intensity for the given earthquake.

Consequently, depending on the number of earthquakes in the sequence, the number of distinct ShakeMap intensity values, and the size of the building stock, an average total number of buildings (*T*) was affected throughout the simulation. All reported results correspond to averages over *N.seeds* = 100 independent runs.

The introduction of the parameter *p* was also motivated by the outcomes obtained when simulating only the mainshock. In that case, the model tended to overestimate the percentages of light-to-severe damage states (D1–D3), while underestimating the proportions of buildings in classes D0, D4, and D5. To identify the value of *p* minimizing the RMSE between the observed final damage distribution (expressed in terms of *D_Global_*) and the simulated distribution at the end of the sequence, we varied *p* from 10% to 100% with increments of 10%. A finer parameter exploration was initially avoided because of computational and memory constraints. For the L’Aquila 2009 case study, the minimum RMSE value (2.52) was obtained for *p* = 20%.

Although the preliminary results showed good agreement with the observed damage distribution, several limitations of the model were identified, suggesting the need for further investigation to improve both robustness and generalizability. In addition to the issues already discussed in [37], the present study introduces an optimized implementation of the Python v3 code, significantly reducing memory requirements and computational time. This improvement allowed a more extensive analysis of the role played by the parameter *p*, as well as of the potential influence of the number of earthquakes, their magnitudes, and the size of the exposed building stock.

First, the optimized implementation enabled a continuous optimization of the parameter *p* through the *minimize_scalar* routine of the SciPy library, using the bounded method within the interval 10–100%. Second, we investigated whether *p* could be related to seismic intensity. To this end, the constant parameter *p* was replaced by an intensity-dependent function *p*(*I*), so that the fraction of randomly selected buildings varied according to the local intensity value. Several functional forms were tested, including linear, quadratic, power-law, and sigmoidal relationships. Among them, the linear function *p*(*I*) = *mI* provided the lowest RMSE values, comparable to or better than those obtained with a constant *p*. Consequently, only the results corresponding to the optimized values of the coefficient *m* are presented in this work.

The methodology was first applied to the 2009 L’Aquila sequence as a calibration benchmark. In order to investigate the contribution of foreshocks and to ensure methodological consistency across different applications, all earthquakes with magnitude Mw ≥ 4.0 occurring within the time interval characterized by seismicity rates above the background level were included. As a result, the seismic sequence considered in the present study contains five additional events compared with the dataset adopted in [37]. Following the calibration analysis, the methodology was applied to two additional seismic sequences characterized by different numbers of earthquakes, building stocks, and magnitude distributions, allowing the assessment of its performance under distinct seismic conditions.

## 3. New Results on the L’Aquila 2009 Earthquake Case Study

**Figure 1:**
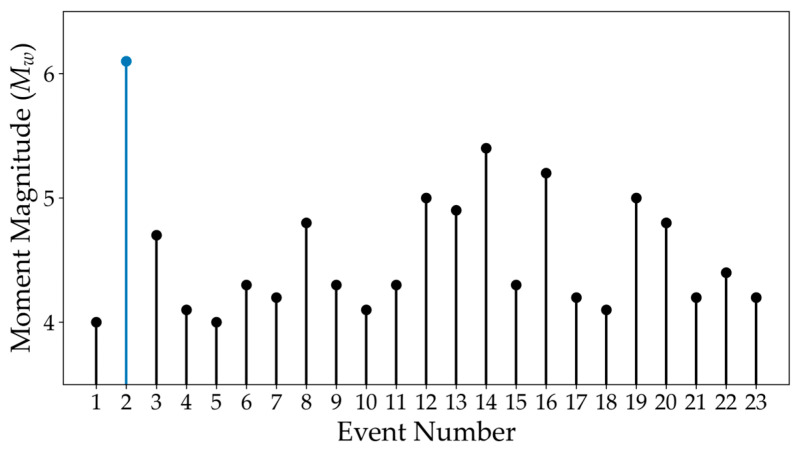
The L’Aquila 2009 seismic sequence selected in this study. In blue, the mainshock. Data from [40].

As discussed in the previous section, the seismic sequence adopted for the L’Aquila 2009 damage scenario includes the Mw 4.0 foreshock of 30 March 2009, together with four additional aftershocks that occurred during April, June, and July 2009. Figure 1 shows the seismic sequence used as input in the present study. The sequence comprises one Mw 4.0 foreshock (EN 1), the Mw 6.1 mainshock (EN 2), four Mw ≥ 5.0 aftershocks (ENs 12, 14, 16, and 18), four events with 4.5 ≤ Mw < 5.0 (ENs 3, 8, 13, and 20), and thirteen earthquakes with 4.0 ≤ Mw < 4.5. Further details on the seismotectonic setting and on the characteristics of the building stock can be found in [37] and the references therein.

**Figure 2:**
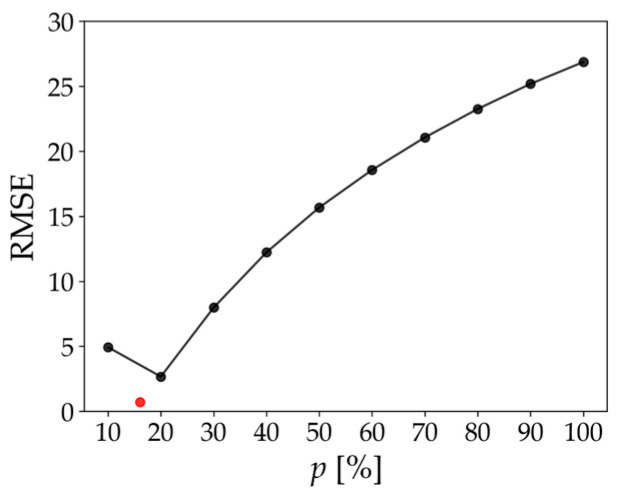
RMSE values between observed and simulated final percentages against parameter *p*, for the L’Aquila 2009 case study (23 events of Mw ≥ 4.0). The black dots represent the values obtained by varying *p* from 10% to 100% with a step of 10%. The minimum RMSE value (2.67) is reached at *p* = 20%. The red dot represents the minimum RMSE value (0.71) found at *p* = 16.06% with the optimization procedure.

As a first step, we simulated the L’Aquila 2009 damage scenario by varying the parameter *p* from 10% to 100% in increments of 10%. The minimum RMSE value obtained was 2.67, corresponding to *p* = 20%. As illustrated in Figure 2, these results are consistent with those previously obtained for the 18-event scenario analyzed in [37], although the minimum RMSE is slightly higher in the present case. Subsequently, by applying the optimization procedure described in Section 2, we identified a substantially lower minimum RMSE value of 0.71 at *p* = 16.06% (red marker in Figure 2). This marked reduction in the error was accompanied by a significantly improved agreement between simulated and observed damage distributions, particularly for the percentages of buildings belonging to the D0 and D5 damage classes.

**Figure 3:**
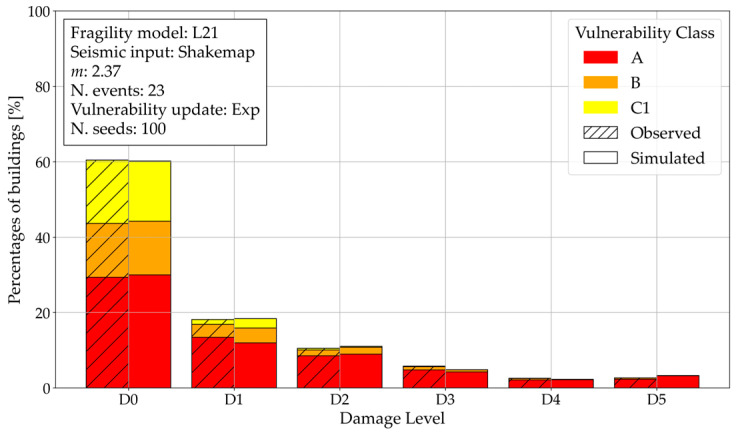
Comparison between the distribution of final total damage levels simulated (solid bars) for the L’Aquila 2009 seismic sequence scenario (23 events of Mw ≥ 4.0), for *m* = 2.37, and the observed distribution from the Da.D.O. [30,31] processed according to the global damage (diagonally hatched bars). The simulations have employed the L21 fragility model and the exponential update vulnerability rule “Exp”. For each damage level, stacked bars also illustrate the distribution of the initial vulnerability classes of the buildings: A (red), B (orange), and C1 (yellow).

We then replaced the constant parameter *p* with the intensity-dependent function *p*(*I*) = *mI*, so that the fraction of buildings randomly sampled during each event became a function of the local seismic intensity. In this case, the optimization procedure was performed with respect to the parameter *m*. For the L’Aquila 2009 scenario, the minimum RMSE value obtained was 0.73 for *m* = 2.37. Compared with the optimized constant-parameter case (*p* = 16.06%), no substantial differences were observed in the final damage distribution. Figure 3 presents the average distribution of final damage levels obtained over 100 simulation runs for the optimal value *m* = 2.37.

**Figure 4:**
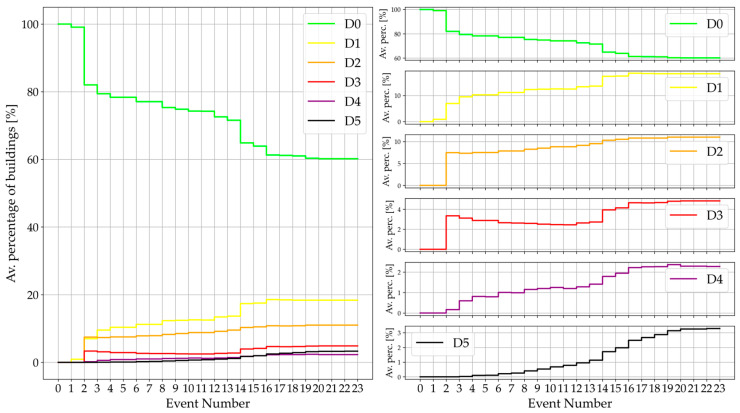
Temporal evolution of the averaged (over 100 runs) percentages of building in each damage level from D0 (undamaged) to D5 (collapsed), for *m* = 2.37. The left panel provides an overall comparison of the evolution of all damage levels in a single plot, while the right panel presents each damage level separately to highlight incremental changes.

In the present study, we also investigated the temporal evolution of cumulative damage throughout the seismic sequence. To this end, we analyzed the average percentage of buildings in each damage class (D0–D5) after every event, considering the mean values over 100 simulation runs. As shown in Figure 4, for *m* = 2.37, the mainshock (EN 2) causes approximately 20% of the buildings to transition from the undamaged state (D0) to the light (D1) and moderate (D2–D3) damage classes, with a smaller increase also observed in D4. During the subsequent evolution of the sequence, the percentages of buildings in D1 and D2 continue to increase steadily. In contrast, the proportion of buildings in D3 gradually decreases, while the fractions belonging to the higher damage classes D4 and D5, although relatively small in absolute terms, exhibit a progressive increase. Following the Mw 5.4 aftershock (EN 14), a more pronounced increase is observed across all damage states. A similar pattern emerges for the optimized constant value *p* = 16.06%, with only minor quantitative differences.

These results indicate that, although the mainshock exerts the dominant influence on the overall damage evolution, it does not appear to be solely responsible for the highest damage states (D4 and D5). Rather, these severe damage levels emerge primarily from the progressive deterioration of buildings that had already experienced moderate damage following the mainshock and were subsequently affected by additional earthquakes. On the one hand, this behaviour may suggest a limitation of the model, which could underestimate the direct contribution of the mainshock to severe structural damage. On the other hand, the observed evolution is fully consistent with the cumulative damage hypothesis underlying the methodology: while individually less intense, successive earthquakes progressively weaken already damaged structures, eventually driving them beyond the thresholds associated with the most severe damage states, thus introducing memory effects and path-dependent dynamics.

The results obtained in the L’Aquila 2009 case show that, within the investigated range of intensities and for the considered building stock, the introduction of an intensity-dependent sampling parameter improves the overall performance of the methodology with respect to the preliminary results presented in the previous study [37]. Nevertheless, the outcomes remain substantially equivalent to those obtained using the optimized constant *p* value. In fact, given the maximum and minimum intensities from the ShakeMaps associated with the L’Aquila 2009 sequence, *I_min_* = 1.0 and *I_max_* = 8.8, the corresponding percentage of sampled buildings associated with these intensity values for *m* = 2.37 are 2.37% and 20.86%, respectively. Since the surveyed buildings are predominantly concentrated in the epicentral areas of each ShakeMap, where the highest intensity values prevail, both the constant *p* and variable *p*(*I*) approaches ultimately involve approximately the same fraction of buildings. Consequently, only limited differences emerge between the two modelling strategies.

## 4. Case Study 1: The Garfagnana-Lunigiana 2013 Seismic Sequence

To evaluate the generalizability of the proposed methodology and investigate its possible dependence on the characteristics of the calibration case study, such as the number of earthquakes with Mw ≥ 4.0 and the magnitudes of the mainshock, foreshocks, and aftershocks, we selected a seismic sequence characterized by both a smaller number of events and lower magnitudes. This additional application also offered the opportunity to assess whether the observed damage distribution available in the Da.D.O. could reasonably be interpreted as the cumulative outcome of the entire sequence or mainly as the consequence of the mainshock alone.

Among the seismic sequences available in the Da.D.O. that met these criteria were Pollino 1998 (Mw 5.6 mainshock and no additional event with Mw ≥ 4.0 [41]), Emilia 2003 (Mw 4.7 mainshock followed by one aftershock with Mw ≥ 4.0 [42]), and Garfagnana–Lunigiana 2013 (Mw 5.1 mainshock followed by three aftershocks with Mw ≥ 4.0). The latter was selected because it allows the cumulative damage process to be analyzed over more than two events and, importantly, because the macroseismic survey adopted the same damage assessment form used for the L’Aquila 2009 sequence, ensuring greater consistency in the comparison despite the overall objective of Da.D.O. being the provision of homogeneous damage datasets.

### 4.1. The Garfagnana-Lunigiana 2013 Seismic Sequence

**Figure 5:**
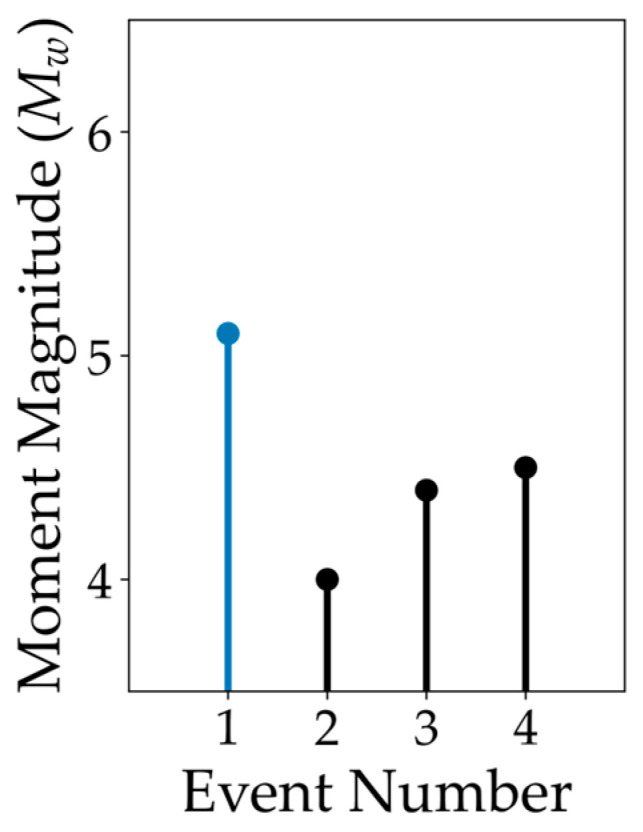
The Garfagnana-Lunigiana 2013 seismic sequence. In blue, the mainshock followed by three events with Mw ≥ 4.0. Data from [40].

The seismic sequence shown in Figure 5 consisted of a Mw 5.1 mainshock on 21 June 2013, followed by three aftershocks with magnitudes exceeding Mw 4.0. According to INGV records, more than 2400 earthquakes occurred between 15 June and the end of the following month, with seismicity extending northeastward from the mainshock epicentre [40]. Although the mainshock magnitude was moderate, the earthquake was felt over a wide area of northern and central Italy. The epicentral region is located in northern Tuscany, encompassing parts of the provinces of Pistoia, Massa-Carrara, and Lucca, historically known as Garfagnana and Lunigiana.

The focal mechanism of the June 2013 mainshock, characterized by normal dip-slip faulting on a low-angle (40–50°) NNW-dipping fault plane with a minor right-lateral strike-slip component [43,44,45], provided valuable insight into the complex seismotectonic setting of this sector of the Northern Apennines, situated along the northern margin of the Apuan Alps. The area is affected by an extensional tectonic regime associated with the development of intramontane basins and the rollback of the Adriatic plate, with normal faults generally trending NW-SE, dipping toward the SW or NW, and frequently organized in segmented systems interconnected by transfer zones. Seismicity is characterized by both deep (>35 km) and shallow (≤35 km) hypocentres [39].

The area located between the Lunigiana and Garfagnana extensional basins represents the northwestern continuation of the Etrurian Fault System [46]. Most of the historical seismic activity has occurred within this transfer zone—often referred to as a “land of earthquakes”—where damaging earthquakes (Is > 6 MCS) have occurred on average every thirty years between 1976 and 2013 [47]. The strongest event in the region was the 7 September 1920 earthquake (Mw 6.5, Imax = 10 MCS), which played a key role in the development of seismic prevention policies. Following that event, the affected area was included among those subject to specific seismic regulations (Royal Decree Law n. 1315, 23 September 1920), introducing restrictions on construction, including no-build zones, maximum building heights, and prescribed construction techniques and materials. These provisions were subsequently incorporated into the national seismic classification system through Royal Decree n. 431 of 13 March 1927, which designated the area as seismic zone 2. More recently, after the October 1995 earthquake, the Tuscany Regional Law n. 56 of 30 July 1997 promoted further risk mitigation and prevention measures in seismically classified municipalities.

As discussed in the following sections, the combination of a well-documented seismic history and the progressive implementation of mitigation policies may have contributed to limiting the severity of damage observed during the 2013 sequence [47].

### 4.2. The Macroseismic Survey and the Observed Damage

The QUEST macroseismic team surveyed 27 localities where macroseismic intensities exceeded 5 MCS-EMS [47]. The survey focused primarily on external damage affecting residential buildings. Most of the investigated settlements consist of historic medieval centres dominated by stone masonry buildings, mainly belonging to vulnerability classes A and B, often characterized by poor maintenance conditions and partial abandonment. These coexist with more recent reinforced-concrete and reinforced-masonry buildings constructed after 1920 and after 1995.

The highest damage levels were recorded in the oldest masonry buildings, whereas the newer structures generally exhibited only slight damage. The survey report also documents the effects of the Mw 4.4 aftershock of 23 June, which caused a D4 damage level (roof structural failure) in a class A building located in the village of Metra, within the municipality of Minucciano.

**Figure 6:**
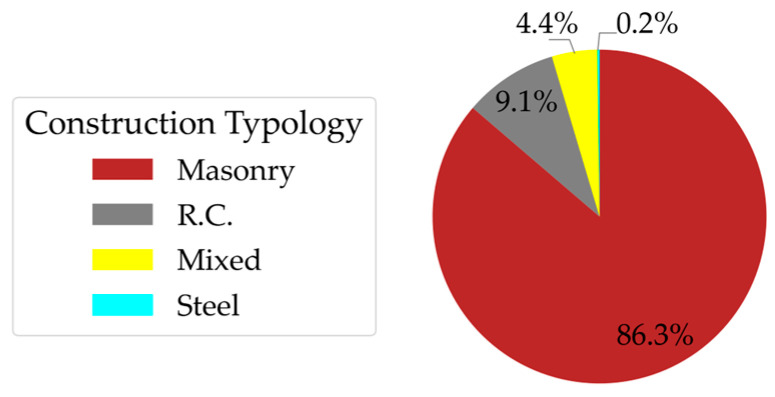
Percentages of construction types of buildings in the Da.D.O. Garfagnana-Lunigiana 2013 dataset [30,31].

The QUEST survey report [47] constitutes the reference source for the Garfagnana–Lunigiana 2013 dataset included in the Da.D.O. database [30,31]. The original dataset contains information on 3258 residential buildings distributed across 20 municipalities within the provinces of Lucca, Pistoia, and Massa-Carrara, and is overwhelmingly dominated by masonry constructions (Figure 6).

Using the Completeness Ratio (CR) proposed in [11] and comparing the surveyed buildings with the ISTAT 2011 census data [48], only one municipality, Casola in Lunigiana, was found to have a survey coverage exceeding 50% of its building stock (CR ≈ 74%).

**Figure 7:**
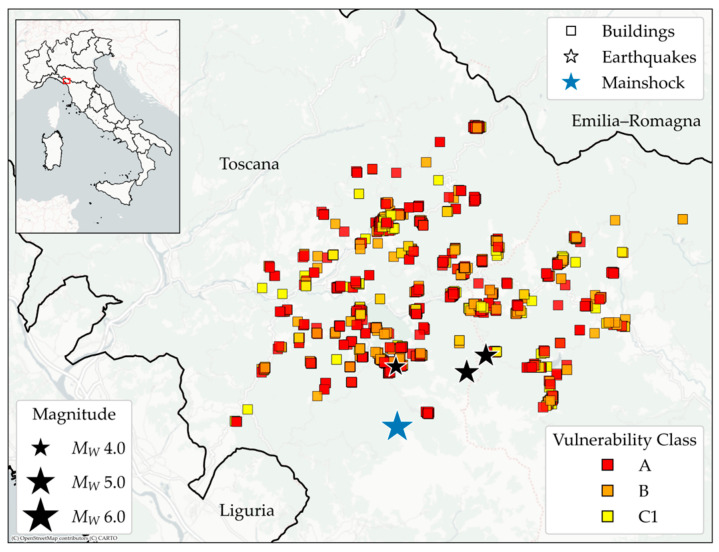
Spatial distribution of the 2544 masonry buildings selected after preprocessing from the Da.D.O. original Garfagnana-Lunigiana 2013 dataset [30,31]. Buildings are represented as squares coloured according to their initial vulnerability class, ranging from A (most vulnerable) to C1 (least vulnerable). The stars indicate the epicentres of the four earthquakes with magnitude Mw ≥ 4.0, with symbol size proportional to magnitude; the blue star denotes the mainshock. The data on the earthquakes are from [40]. The inset map shows the location of the study area, highlighted by a red box, within the Italy territory.

Following the same preprocessing procedure adopted for the L’Aquila 2009 case study, we selected only masonry buildings for which both observed damage data and the structural information required to assign a vulnerability class were available. After preprocessing, the operative dataset consisted of 2544 masonry buildings. Figure 7 illustrates their spatial distribution, with buildings coloured according to vulnerability class and superimposed on the epicentral locations of the earthquakes composing the sequence.

### 4.3. Results on the Garfagnana-Lunigiana 2013 Case Study

In this section, we present the results obtained for the Garfagnana-Lunigiana 2013 case study by comparing the two formulations of the methodology: first using a constant value of *p*, and subsequently adopting an intensity-dependent parameter *p*(*I*).

**Figure 8:**
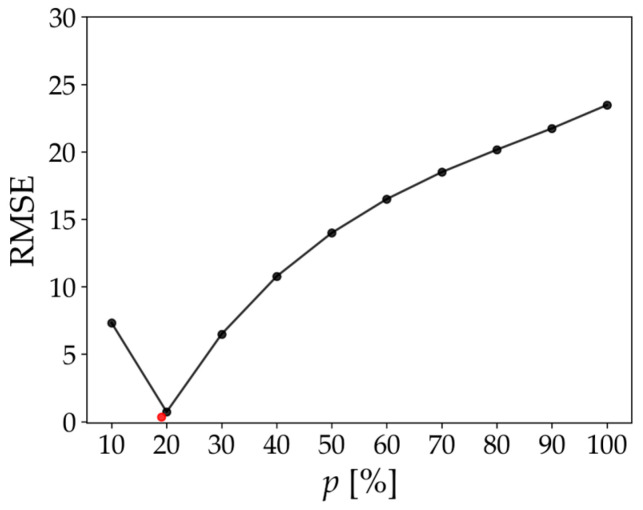
RMSE values between observed and simulated final percentages against the parameter *p* for the whole sequence scenario of the Garfagnana-Lunigiana 2013 case study. The black dots represent the values obtained by varying the value of *p* from 10% to 100% with a step of 10%. The minimum RMSE value (0.72) is reached at *p* = 20%. The red dot represents the minimum RMSE value (0.34) found at *p* = 19.06%.

Following the same procedure used for the L’Aquila 2009 calibration case, we initially simulated the Garfagnana-Lunigiana sequence by varying *p* between 10% and 100% in increments of 10%. The minimum RMSE obtained through this discrete exploration was 0.72, corresponding to *p* = 20%. Applying the optimization procedure described previously further reduced the RMSE to 0.34, achieved at *p* = 19.06%. Figure 8 shows the RMSE values obtained from the discrete exploration (black markers) together with the optimized minimum value (red marker). The overall trend closely resembles that observed for the L’Aquila 2009 case study (Figure 2).

We then applied the optimization procedure to the intensity-dependent formulation *p*(*I*) = *mI*. In this case, the minimum RMSE value obtained was 0.32 for *m* = 3.01. Considering the ShakeMap intensity range associated with the Garfagnana–Lunigiana sequence (*I_min_* = 3.6 and *I_max_*= 7.2), the corresponding percentages of sampled buildings vary between 10.84% and 21.67%, respectively.

**Figure 9:**
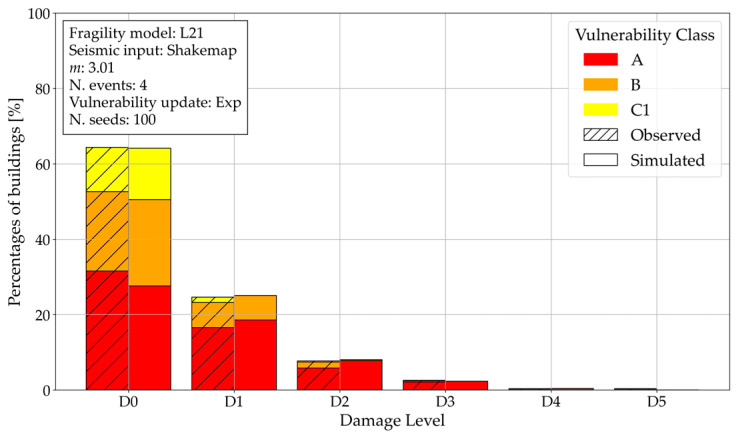
Comparison between the distribution of final total damage levels simulated (solid bars) for the Garfagnana-Lunigiana 2013 case study, for *m* = 3.01, and the observed distribution from the Da.D.O. [30,31] processed according to the global damage (diagonally hatched bars). The simulations have employed the L21 fragility model and the exponential update vulnerability rule “Exp”. For each damage level, stacked bars also illustrate the distribution of the initial vulnerability classes of the buildings: A (red), B (orange), and C1 (yellow).

Figure 9 presents the final damage distribution obtained for *m* = 3.01. In contrast to the L’Aquila case, the differences among the results obtained using *p* = 20%, *p* = 19.06%, and *m* = 3.01 are practically negligible, both in terms of the final damage distribution and of the temporal evolution of damage. The only appreciable difference between the two constant *p* simulations is a slight increase in the proportion of class A buildings remaining in D0, accompanied by a corresponding decrease in classes D1 and D2.

**Figure 10:**
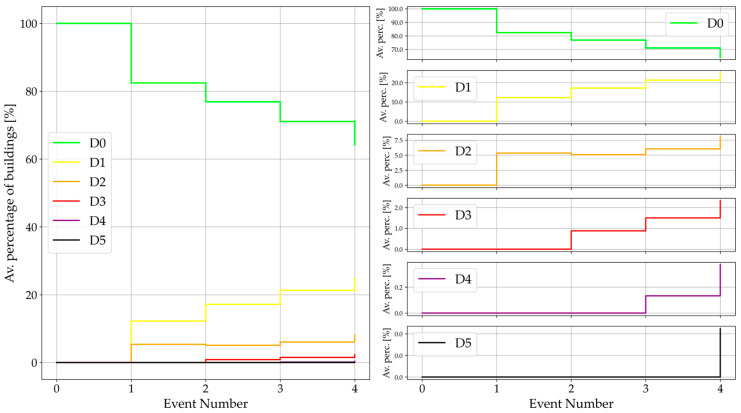
Temporal evolution of the averaged (over 100 runs) percentages of building in each damage level from D0 (undamaged) to D5 (collapsed), for *m* = 3.01. The left panel provides an overall comparison of the evolution of all damage levels in a single plot, while the right panel presents each damage level separately to highlight incremental changes.

The temporal evolution of damage, averaged over 100 simulation runs, is shown in Figure 10. For *m* = 3.01, the mainshock causes fewer than 20% of the buildings to transition from the undamaged state (D0) to damage classes D1 and D2. The second event (Mw 4.0) further increases the proportion of buildings in D1 and produces the transition of a very small number of buildings from D2 to D3.

The third event (Mw 4.4) leads to an increase in all damaged states up to D4, while the fourth event (Mw 4.5) produces the first simulated collapse (D5), involving less than one building on average. It is important to recall that the observed damage distribution used for comparison is assumed to represent the final state reached at the end of the seismic sequence. Regarding the actual damage progression, the macroseismic survey documents only one building collapse, which occurred after the third event [47]. In comparison, the model reproduces a marked worsening of damage following the third earthquake but predicts a collapse only after the fourth event.

More generally, the simulations suggest that the mainshock was primarily responsible for slight-to-moderate damage, whereas the subsequent earthquakes played a crucial role in driving buildings toward the higher damage classes. This behaviour is consistent with the cumulative damage framework adopted in the present study and highlights the importance of considering the entire seismic sequence when interpreting observed damage patterns.

## 5. Case Study 2: The Central Italy 2016–2017 Sequence

As a second validation case, we applied the proposed methodology to the 2016–2017 Central Italy seismic sequence, a significantly more complex scenario than both the calibration case (L’Aquila 2009) and the Garfagnana-Lunigiana 2013 sequence. This sequence is characterized by a larger number of earthquakes, higher magnitudes, and a substantially broader geographical extent, involving a much larger building stock.

Among the datasets available in the Da.D.O. database, both the Irpinia 1980 and the Central Italy 2016–2017 earthquakes provide extensive post-event damage information. The latter was selected because of the exceptional complexity of the seismic sequence and, consistently with the previous case studies, because the same AeDES survey form was employed for damage assessment, ensuring methodological homogeneity across the datasets.

### 5.1. The Central Italy 2016–2017 Sequence

The Central Italy seismic sequence extended from August 2016 to December 2017 and represents one of the most significant seismic crises in recent Italian history. The sequence culminated with the Mw 6.5 Norcia earthquake of 30 October 2016, the strongest event recorded in Italy since the Mw 6.9 Irpinia earthquake of 1980. The mainshock was preceded by a complex foreshock activity, including the Mw 6.0 Amatrice earthquake of 24 August 2016, two Mw 5.4 events, and a Mw 5.9 earthquake on 26 October 2016, and was followed by an extended aftershock sequence including four earthquakes with Mw ≥ 5.0.

**Figure 11:**
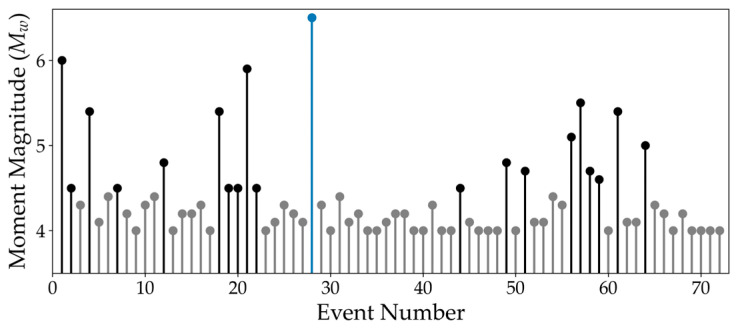
The Central Italy seismic sequence. The earthquakes are coloured based on their magnitude: in grey 4.0 ≤ Mw < 4.5, in black 4.5 ≤ Mw < 6.0, in blue the Mw 6.5 mainshock. Data from [40].

Figure 11 summarizes the 72 earthquakes with magnitude Mw ≥ 4.0 that occurred between August 2016 and December 2017. The August 24 event alone caused widespread destruction and 299 fatalities, while both the Amatrice and Norcia earthquakes generated significant surface ruptures [49,50].

The sequence developed within the extensional tectonic framework of the central Apennines, between the source regions of the Umbria-Marche 1997–1998 and L’Aquila 2009 earthquakes. The activated fault system consisted of multiple interacting fault segments extending over more than 80 km [51,52]. Following the Amatrice earthquake, seismicity progressively migrated northwest toward the Norcia area, where the Mw 6.5 mainshock occurred, before propagating southeast toward the Campotosto area during January 2017.

The affected territory is characterized by numerous small historical settlements located in mountainous areas and composed predominantly of masonry buildings. These structures often exhibit construction features associated with high seismic vulnerability, including irregular stone masonry, poor wall connections, rubble infill, and traditional timber floors and roofs. Historical records indicate that many of these settlements have repeatedly experienced destructive earthquakes, including the 1627, 1639, and 1703 events, which severely damaged or destroyed several of the towns later affected during the 2016–2017 sequence.

Although seismic regulations had been progressively introduced throughout the twentieth century, the damage observed during the sequence revealed the persistence of significant structural vulnerability. In this context, Valensise et al. [7] introduced the concept of forgotten vulnerability, referring to the gradual loss of seismic awareness and mitigation practices in communities where long periods have elapsed since the last destructive earthquake. Such a process may lead to inadequate maintenance, insufficient retrofitting, and the adoption of construction modifications that inadvertently increase vulnerability, such as the replacement of lightweight timber floors with heavy reinforced concrete slabs without appropriate strengthening of masonry walls.

### 5.2. The Macroseismic Survey and the Observed Data

The Central Italy sequence represents a unique case in the Italian seismic record because, for the first time, damage surveys were systematically updated after each major damaging event [34]. Nevertheless, despite the availability of modern survey protocols and extensive field operations, reconstructing the temporal evolution of damage over such a large territory remained a challenging task.

The first survey phase followed the Mw 6.0 Amatrice earthquake of August 2016. Rapid assessments were initially conducted using the MCS scale [53], while parallel surveys based on the EMS-98 methodology allowed a more detailed evaluation of the relationship between building vulnerability and observed damage [54]. In the epicentral area, intensities reached I = 10 EMS. The most vulnerable masonry buildings frequently suffered severe damage or collapse, while reinforced concrete structures generally experienced damage concentrated in non-structural elements. Evidence of cumulative effects was already reported during this phase, particularly in localities such as San Pellegrino di Norcia, where the Mw 5.4 earthquake of the same day contributed to the worsening of damage conditions.

The second survey phase followed the seismic crisis of late October 2016, culminating in the Mw 6.5 Norcia earthquake. The combination of the Mw 5.4 and Mw 5.9 events of 26 October and the Mw 6.5 mainshock of 30 October dramatically worsened the damage scenario already produced in August and extended severe damage to areas north of Norcia. According to the updated QUEST reports [55], increases of up to three EMS intensity degrees were documented in several localities. In Accumoli, for example, areas initially assigned intensity I = 8 EMS after the August events reached intensity I = 10 EMS after the October sequence, with near-total destruction affecting a large fraction of the most vulnerable buildings.

A third survey phase followed the January 2017 seismic activity. Four earthquakes with Mw ≥ 5.0 produced additional damage in the Campotosto area and surrounding localities, increasing local intensities from approximately 5 EMS to 8 EMS [56,57].

**Figure 12:**
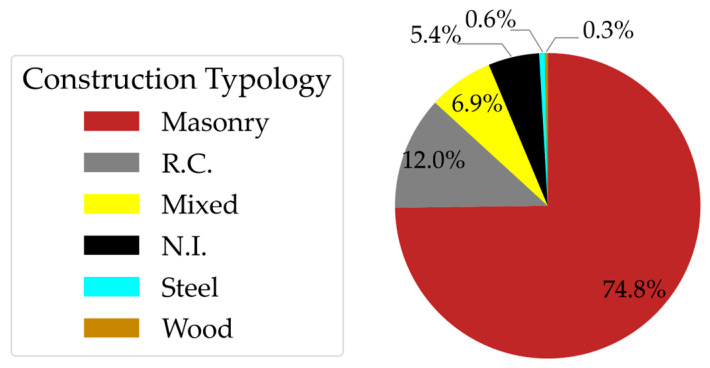
Percentages of construction types of buildings in the Da.D.O. Central Italy 2016–2017 dataset [30,31].

The Da.D.O. dataset associated with this sequence contains information on 89,268 buildings distributed across 481 municipalities in the regions of Marche, Umbria, Lazio, and Abruzzo. As shown in Figure 12, masonry buildings account for approximately three-quarters of the entire dataset.

**Figure 13:**
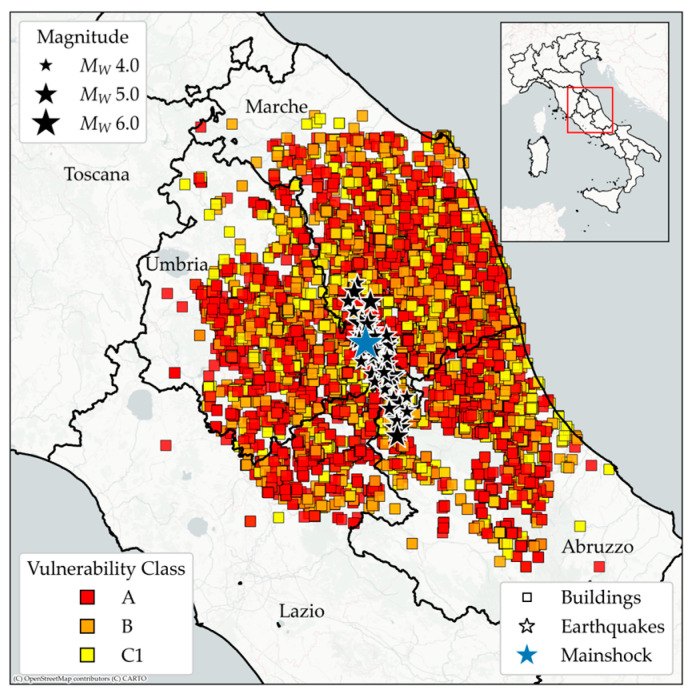
A georeferenced map of the 65,164 buildings (squares) of the processed data from the Central Italy 2016–2017 dataset in Da.D.O. [30,31]. Buildings are represented as squares coloured according to their initial vulnerability class, ranging from A (most vulnerable) to C1 (least vulnerable). The stars indicate the epicentres of the 72 earthquakes with Mw ≥ 4.0 recorded from August 2016 to December 2017, with symbol size proportional to magnitude; the blue star denotes the mainshock. The data on the earthquakes are from [40]. The inset map shows the location of the study area, highlighted by a red box, within the Italy territory.

Following the same preprocessing procedure adopted in the previous case studies, we retained only masonry buildings for which complete structural and damage information was available. The resulting operative dataset includes 65,164 buildings. Their spatial distribution, together with the epicentral locations of the 72 earthquakes considered in this study, is shown in Figure 13.

### 5.3. Results on the Central Italy 2016-2017 Case Study

We now discuss the results obtained for the Central Italy 2016–2017 sequence. As in the previous analyses, simulations were first performed assuming a constant value of the parameter *p*, and subsequently using the intensity-dependent formulation *p*(*I*).

**Figure 14:**
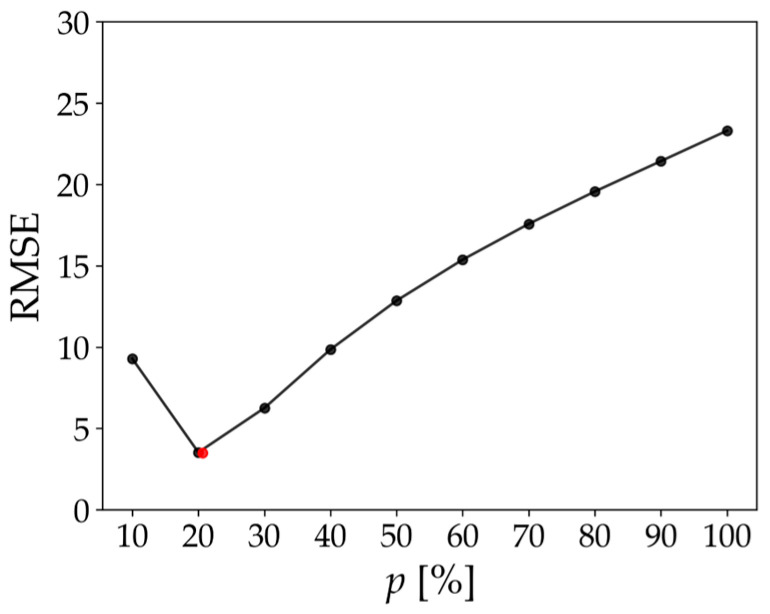
RMSE values between observed and simulated final percentages against the parameter *p* for the whole sequence scenario of the Central Italy 2016–2017 case study. The black dots represent the values obtained varying the value of *p* from 10% to 100% with a step of 10%. The minimum RMSE value (3.53) is reached at *p* = 20%. The red dot represents the minimum RMSE value (3.51) found at *p* = 20.65%.

The optimization results show an even stronger consistency between the discrete and continuous calibration procedures than in the previous case studies. The exploration performed with 10% increments identifies a minimum RMSE value of 3.53 for *p* = 20%, while the optimization procedure yields a very similar minimum value of 3.51 at *p* = 20.65% (Figure 14). This remarkable agreement further supports the robustness of the calibration framework.

Applying the intensity-dependent formulation, the minimum RMSE value obtained was 3.74 for *m* = 3.05. Given the ShakeMap intensity range associated with the sequence *I_min_* = 0.0 and *I_max_* = 9.8, the corresponding percentage of buildings involved in the damage updating process varies between 0% and 29.89%.

**Figure 15:**
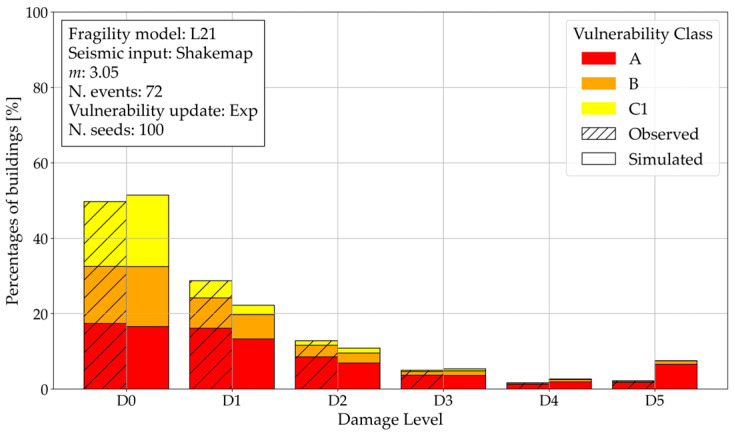
Comparison between the distribution of final total damage levels simulated (solid bars) for the Central Italy 2016–2017 case study, for *m* = 3.05, and the observed distribution from the Da.D.O. [30,31] processed according to the global damage (diagonally hatched bars). The simulations have employed the L21 fragility model and the exponential update vulnerability rule “Exp”. For each damage level, stacked bars also illustrate the distribution of the initial vulnerability classes of the buildings: A (red), B (orange), and C1 (yellow).

**Figure 16:**
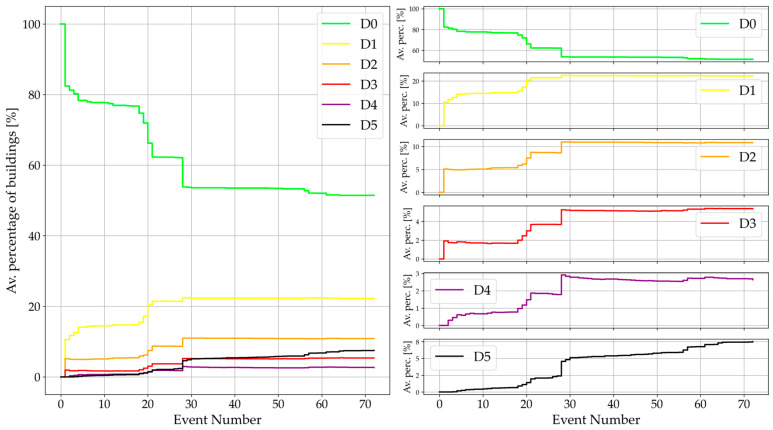
Temporal evolution of the averaged (over 100 runs) percentages of building in each damage level from D0 (undamaged) to D5 (collapsed), for the Central Italy 2016–2017 scenario, for *m* = 3.05. The left panel provides an overall comparison of the evolution of all damage levels in a single plot, while the right panel presents each damage level separately to highlight incremental changes.

The resulting final damage distribution is shown in Figure 15, while Figure 16 reports the temporal evolution of damage throughout the sequence. Compared with the previous case studies, the minimum RMSE values obtained for the Central Italy sequence are significantly larger. In particular, the model tends to underestimate the fractions of buildings experiencing slight damage (D1–D2), while overestimating both the proportion of undamaged buildings (D0) and the percentage of structures reaching moderate-to-severe damage states, especially collapse (D5). A similar tendency was already observed in the L’Aquila case study, although it is more pronounced here.

The temporal evolution of damage provides useful insight into the origin of these discrepancies. Following the first major event (EN 1 in Figure 11, corresponding to the Mw 6.0 Amatrice earthquake), approximately 20% of the buildings leave the undamaged state and transition into damage classes ranging from D1 to D4. Although not visually evident in Figure 16, the fraction of buildings in D4 already increases by about 0.5% after this first event. Subsequent earthquakes progressively amplify the damage scenario in a non-linear way. A first growth phase continues until the Mw 5.4 earthquake identified as EN 4, after which the damage levels temporarily stabilize. A second acceleration occurs between EN 18 (Mw 5.4) and EN 21 (Mw 5.9), producing a further increase in the damaged states. The most significant transition is associated with EN 28, corresponding to the Mw 6.5 Norcia mainshock, after which the damage distribution approaches its final configuration. The remaining events mainly produce incremental changes, with modest additional increases observed after EN 55 (Mw 5.1) and EN 56 (Mw 5.5).

Overall, the simulations clearly reproduce the stepwise accumulation of damage induced by successive strong earthquakes. However, the larger discrepancies with respect to the observed distribution suggest that, for highly complex seismic crises involving numerous large events and strong spatial heterogeneity, additional mechanisms may contribute to the observed damage evolution. These aspects will be further discussed in the final section.

## 6. Discussion

The primary objective of this study was to investigate the influence of several key parameters on the cumulative seismic damage model originally proposed in [37], with particular attention to the role of the parameter *p*, the number of earthquakes composing a sequence, their magnitudes, and the size of the exposed building stock. To this end, we first optimized the computational implementation of the methodology, substantially reducing memory requirements and execution times. We then revisited the L’Aquila 2009 calibration case study and subsequently applied the methodology to two additional seismic sequences characterized by markedly different seismic and urban features.

The comparison among the three case studies provides useful insight into both the strengths and limitations of the proposed approach. Table 2 summarizes the optimal values of the calibration parameters and the corresponding RMSE values obtained for each scenario.

In the L’Aquila 2009 case study, the effect of the number of earthquakes included in the analysis clearly emerges. The introduction of five additional events with Mw ≥ 4.0, adopted to ensure methodological consistency among the three case studies, resulted in slightly higher RMSE values than those obtained for the original 18-event configuration. This outcome suggests that the contribution of smaller earthquakes to cumulative damage may depend on the specific characteristics of the sequence and highlights the need for future investigations aimed at identifying optimal magnitude thresholds for event selection.

The Garfagnana-Lunigiana 2013 case study represents the opposite situation, being characterized by a relatively small number of earthquakes and a much smaller building stock. Although this scenario produced the lowest RMSE values among all analyzed cases, it also exhibited the largest variability among simulation runs, as indicated by the stability analyses (not reported here). As shown in Table 2, the average percentage of buildings affected at least once during the sequence (*T*) is slightly above 50%, whereas in both the L’Aquila and Central Italy case studies nearly all buildings are affected during the simulation. This reduced sampling coverage likely contributes to the larger fluctuations observed between realizations.

In addition, the Garfagnana-Lunigiana simulations tend to underestimate the percentage of the highest damage states (D4 and D5). While this behaviour may partly reflect limitations of the model, it could also be influenced by uncertainties in the seismic input data. As already discussed, available ShakeMaps for this sequence may overestimate the associated intensities, while different seismic catalogues report slightly different values for the mainshock magnitude, ranging from Mw 5.1 to Mw 5.3.

The Central Italy 2016–2017 sequence proved to be the most challenging scenario for the methodology, yielding the largest RMSE values. One possible explanation lies in the exceptional complexity of the sequence itself. Unlike the previous case studies, the Central Italy sequence includes a large number of moderate earthquakes interspersed among a few very strong events. Under these conditions, the strongest earthquakes may dominate the observed damage pattern, while the cumulative contribution of the numerous smaller events may be overestimated by the model, ultimately leading to damage saturation effects.

From a broader perspective, these results suggest that cumulative seismic damage should not be interpreted as a simple additive process. The progressive updating of vulnerability introduces memory into the system, causing the effects of each earthquake to depend on the entire previous seismic history. Consequently, buildings with similar structural characteristics and subjected to similar shaking levels may evolve toward different damage states depending on the sequence of events experienced. This path-dependent behaviour represents one of the key features captured by the proposed methodology.

A more comprehensive assessment of this mechanism would require damage observations collected after individual earthquakes within a sequence. Although such datasets are rare, given the logistical challenges to conducting repeated post-earthquake surveys during sequences, the notable example of the Central Italy 2016–2107 sequence in [34] offers a promising opportunity for future validation of the simulated temporal evolution of damage.

The simulations also highlight the emergence of collective damage patterns at the urban scale. While damage accumulation occurs at the level of individual buildings, the final damage distributions observed across entire urban areas exhibit regularities that cannot be directly inferred from the characteristics of single structures. Such emergent behaviour supports the adoption of a systems-level perspective for seismic risk assessment, complementing traditional building-scale approaches.

In this context, a source of uncertainty concerns the initial conditions of the exposed building stock. In fact, a part of the Campotosto area was still undergoing reconstruction following the 2009 L’Aquila earthquake [57]. Nevertheless, consistent with the assumptions adopted for all case studies, buildings were initialized in an undamaged state at the beginning of the simulations. Under this assumption, one would expect a tendency toward damage underestimation. Interestingly, the opposite behaviour is observed, with an overestimation of the highest damage levels (D4 and D5). This result suggests that the main source of discrepancy is more likely related to the cumulative damage mechanism itself and to the interaction between numerous moderate events and vulnerability updating, rather than to the assumed initial conditions. This is particularly relevant for the Central Italy 2016–2017 case study, whose prolonged and complex succession of damaging events may require further investigation of alternative cumulative damage mechanisms and fragility models.

Overall, the optimized calibration procedure significantly improves the performance of the methodology. The introduction of a continuous search for the optimal value of *p* consistently reduces the RMSE relative to the preliminary analyses presented in [37], particularly for the L’Aquila 2009 and Garfagnana–Lunigiana 2013 case studies. More limited improvements are observed for the Central Italy sequence, reflecting the intrinsic complexity of that seismic crisis.

An additional objective of this work was to investigate whether the parameter *p* could be linked to physically meaningful variables. To this end, we replaced the constant parameter with the intensity-dependent function *p*(*I*) = *mI*, representing the simplest and most straightforward implementation of such a relationship.

Within the proposed framework, *p* represents the fraction of buildings that are randomly selected at each earthquake and intensity level to be potentially affected by the seismic sequence. Damage is not automatically assigned to these buildings but depends on the interaction between local intensity and structural vulnerability. Remarkably, despite the substantial differences among the three case studies, the optimal values of the coefficient *m* consistently converge toward values close to 3. Similar results were obtained for different optimization intervals, while the analyses presented here refer to the range *m* ∈ [1, 10].

This apparent stability suggests that *m* ≈ 3 may represent a robust parameter of the methodology. If confirmed by future applications, such a value could be adopted as a default calibration parameter when observed damage data are unavailable. In this context, the methodology could be employed to generate synthetic but realistic cumulative damage scenarios based on simulated seismic sequences and existing building inventories. However, such use should be limited to regions where the exposed building stock is characterized by masonry typologies and seismicity comparable to those investigated in this study. Its applicability to different construction practices, structural characteristics or seismic hazard should not be assumed and should be verified using local data whenever possible. Further validation using datasets from additional regions and structural typologies is needed to assess its broader transferability. Such applications would significantly extend the practical usefulness of the proposed approach for seismic risk assessment and territorial planning.

## 7. Conclusions

Assessing the cumulative effects of repeated seismic loading on urban building stocks remains one of the most challenging problems in seismic risk analysis. Although the importance of damage accumulation is widely recognized, its quantitative representation at the urban scale is still an active area of research. In this work, we further developed and tested a methodology specifically designed to simulate cumulative seismic damage scenarios generated by real earthquake sequences. Starting from the preliminary results obtained for the 2009 L’Aquila sequence, we introduced an optimized calibration procedure and extended the analysis to the Garfagnana-Lunigiana 2013 and Central Italy 2016–2017 seismic sequences. The results demonstrate that the methodology is capable of reproducing, with reasonable accuracy, the observed distributions of building damage while explicitly accounting for both damage accumulation and the progressive evolution of structural vulnerability. The analyses also indicate that the performance of the model might depend on several factors, including the number of earthquakes in the sequence, their magnitude distribution, and the characteristics of the exposed building stock. The comparison among the three case studies highlights both the potential and the current limitations of the proposed approach. In particular, the methodology performs well for relatively compact seismic sequences, while more complex crises characterized by numerous moderate-to-strong earthquakes may require further refinements in the representation of cumulative damage processes. A noteworthy outcome of this study is the emergence of a nearly constant optimal value of the coefficient governing the intensity-dependent formulation of the parameter *p*. This result suggests the possibility of extending the methodology beyond calibration exercises and applying it to predictive analyses in areas where post-earthquake damage datasets are unavailable. Future developments will focus on the generation of synthetic seismic sequences and on the simulation of cumulative-damage scenarios for urban areas exposed to significant seismic hazard. Such analyses could be performed at both municipal and urban scales and may provide valuable support for seismic risk mitigation strategies, emergency planning, recovery policies, and Civil Protection decision-making processes.

Finally, beyond its practical applications in seismic risk assessment, the proposed methodology contributes to the study of urban systems as complex adaptive systems. The results indicate that memory effects, vulnerability evolution, and cumulative damage accumulation play a fundamental role in shaping post-earthquake damage patterns. Future developments may therefore benefit from integrating concepts and tools from complexity science, including the analysis of emergent behaviours, resilience dynamics, and long-term vulnerability evolution under repeated perturbations.

## Figures and Tables

**Table 1:** Vulnerability classes and subclasses of masonry buildings, assigned according to the specific combination of structural features (adapted from [31]) and the plausible ranges of initial vulnerability (adapted from [13]). See also [37].

Vulnerability Class	Vertical Structure	Horizontal Structure	Chains	Plausible Range of Initial Vulnerability *V*_0_
A	Bad quality masonry	Vaults without chains, vaults with chains, deformable slab, semi-rigid slab, unidentified	No	0.95–1.05
	Bad quality masonry	Vaults without chains, unidentified	Yes	
	Good quality masonry	Vaults without chains, vaults with chains, deformable slab, unidentified	No	
B	Bad quality masonry	Rigid slab	No	0.75–0.85
	Bad quality masonry	Vaults with chains, deformable slab, semi-rigid slab, rigid slab	Yes	
	Good quality masonry	Semi-rigid slab	No	
	Good quality masonry	Vaults without chains, vaults with chains, deformable slab, unidentified	Yes	
C1	Good quality masonry	Rigid slab	No	0.60–0.65
	Good quality masonry	Semi-rigid slab, rigid slab	Yes	

**Table 2:** Summary of the values of *p*, *m*, RMSE and *T* for the three case studies.

Case Study	Parameter	RMSE	T
L’Aquila 2009	*p* = 20%	2.67	99.4
	*p* = 16.06%	0.71	98.2
	*m* = 2.37	0.73	90.8
Garfagnana-Lunigiana 2013	*p* = 20%	0.72	59.0
	*p* = 19.06%	0.34	57.0
	*m* = 3.01	0.32	55.1
Central Italy 2016–2017	*p* = 20%	3.53	100
	*p* = 20.65%	3.51	100
	*m* = 3.05	3.74	99.9

## Data Availability

The data on the earthquake sequences are available in the Italian Seismological Instrumental and Parametric Database (ISIDe) (https://terremoti.ingv.it/iside, accessed on 3 December 2025, ref. [40]); the ShakeMaps are available in the INGV ShakeMap portal (https://shakemap.ingv.it/, accessed on 24 April 2026, ref. [39]); the building stocks are available in the Database of Observed Damage (Da.D.O.) web-based GIS platform of the Italian Civil Protection Department, developed by the Eucentre Foundation (http://egeos.eucentre.it/danno_osservato/web/danno_osservato, accessed on 16 February 2024, ref. [30,31]). The raw data supporting the conclusions of this article will be made available by the authors on request.
